# Socio-economic and demographic factors influencing open defecation in Haiti: a cross-sectional study

**DOI:** 10.1186/s12889-022-14619-2

**Published:** 2022-11-23

**Authors:** Bénédique Paul, David Jean Simon, Ann Kiragu, Woodley Généus, Evens Emmanuel

**Affiliations:** 1 Department of Agro-socio-economics, Chibas, Université Quisqueya, Port-au-Prince, Haiti; 2 Groupe d’Etude sur les Sciences de la Durabilité, Université Quisqueya, Port-au-Prince, Haiti; 3 Bureau d’Etudes et de Recherche en Statistiques Appliquées, Suivi et Evaluation (BERSA-SE), Port-au-Prince, Haiti; 4 Espace universitaire One Health, Université Quisqueya, Port-au-Prince, Haiti

**Keywords:** Open defecation, Sanitation, Socio-economic, Demographic, Factors, HDHS, Haiti

## Abstract

**Background:**

Open defecation (OD) remains an important public health challenge in Haiti. The practice poses a significantly high risk of disease transmission. Considering these negative health consequences, this paper aims to identify socio-economic and demographic factors that influence OD practice among households in Haiti.

**Methods:**

The study used secondary data from 13,405 households from the Haiti Demographic and Health Survey 2016-2017. Descriptive statistics and bivariate analysis were used to find the preliminary results. Further, multivariate analysis was performed to confirm the findings.

**Results:**

Around one quarter (25.3%) of Haitian households still defecate in the open, almost 10% in urban areas, and nearly 36% in rural areas. Multivariate analysis revealed that the age and sex of the household head, household size, number of children aged 1-14 years old in the household, education level, wealth index, access to mass media, place of residence, and region were significant predictors of OD practice among households in Haiti.

**Conclusion:**

To accelerate the elimination of OD by 2030 and therefore achieve sustainable open defecation-free status, the government of Haiti and its partners should consider wealth disparities among regions and mobilize mass media and community-based networks to raise awareness and promote education about sane sanitation practices. Furthermore, because the possibilities to build toilets differ between rural and urban areas, specific interventions must be spearheaded for each of these regions. The public program can subsidize individual toilets in rural areas with room to collect dry excreta for the preparation of fertilizers, while in urban areas collective toilets can be built in slums. Interventions should also prioritize households headed by women and young people, two underpriviledged socioeconomic groups in Haiti.

## Introduction

Open defecation (OD), defined as the disposal of human feces in fields, forests, bushes, open bodies of water, beaches, or other open spaces [1], remains a major health-related challenge in low- and middle-income countries. Due to its adverse health impacts, the international community has taken action to eliminate the practice of defecating in the open. In 2010, the United Nations General Assembly adopted a resolution in which it recognized access to safe drinking water and sanitation as a fundamental right, essential for the full enjoyment of life and the exercise of all human rights [2]. Subsequently, the adoption of the 2015 target 6.2 of the Sustainable Development Goals (SDGs), called for ending open defecation and achieving universal access to adequate and equitable sanitation [3]. Currently, approximately 494 million people worldwide still defecate in the open [1]. This practice however varies significantly among geographic regions: in Europe for instance, less than 1% of people defecate in the open compared to 18% in Africa and about 2% in Latin America and the Caribbean (LAC) [1]. In Haiti, nearly 40% of the population use pit latrines with slab, and approximately 20% use pit latrines without slab/open pit [4]. Also, estimates show that more than 20% of the population continues to practice OD, the highest proportion in the LAC region [5], exposing the country to the risk of negative health consequences related to the behavior.

The practice of OD generates direct and interactive contaminations of the three environmental compartments: soil, water, and air, exposing human and animal populations to the etiological agents of infectious waterborne diseases and/or intestinal parasitic infections [6]. Studies have shown that OD is associated with several adverse public health outcomes, contributing to the heavy burden of disease worldwide [6–8]. The practice is the leading cause of infectious excreta-related diseases, such as cryptosporidiosis, cholera, and typhoid, among others, as well as soil-transmitted helminthiasis infections which have chronic effects [9–11]. Diseases linked to environmental contamination by microorganisms are numerous in developing countries, especially those caused by bacteria and protozoa transmitted by water [12]. Cholera, for instance, is a major cause of diarrhea and a leading cause of death among children under-five years in developing countries [13–15]. Several comprehensive disease burden studies, focusing mainly on diarrheal diseases stress that inadequate drinking water, sanitation, and hygiene are important risk factors [8, 13, 16]. In Haiti, for instance, intestinal nematodes are frequent [17, 18], transmitted through fecal contamination of the environment, they have been attributed to intestinal blood loss leading to iron deficiency anemia and protein malnutrition in developing countries [6, 15].

Almost all studies on OD focusing on South Asia and Africa have identified various socio-cultural and socio-demographic factors to be key drivers for this phenomenon. Sociocultural barriers have posed a great challenge in improving sanitation facilities in developing countries [19]. Because social processes have an impact on individual-level behaviors [20], studies conducted in India, Nepal, and sub-Saharan Africa have found strong associations between sociocultural norms and OD [21–23]. These factors reflect a variety of determinants, such as gender norms of latrine use [21], preferences to defecate in the open instead of using a latrine [24], or cultural beliefs [22]. Furthermore, socio-demographic factors such as age [25], household wealth status [26, 27], household size [22], and education of the household head [28] have been associated with OD. In addition, almost all of the studies have found that defecating in the open occurs predominantly in rural environments [27, 29, 30].

In Haiti, access to water and sanitation remains the lowest in the Western Hemisphere and the issue of OD persists leaving Haitians vulnerable to disease [1]. Despite concerted efforts to promote sanitation, achieving this goal was complicated by the 2010 earthquake which hit the country killing an estimated 230,000 people, injuring 300,000, and greatly degrading sanitary infrastructure [31, 32]. Consequently, the low levels of sanitation services contributed to the severity and rapid spread of the cholera epidemic in 2010 resulting in 8494 deaths [33]. Notwithstanding the various challenges, Haiti has managed to discontinue cholera transmission since early 2019, even though persisting vulnerabilities remain [34]. To contribute to eliminating OD in the country, it is of paramount importance to understand the potential determinants of the practice in the country. While a substantial body of literature in developing countries has highlighted the importance of factors in predicting the practice of OD, no attention has been given to Haiti. Responding to this need, we build on prior research by examining the socio-economic and demographic factors influencing the practice of OD in Haiti.

## Materials and methods

### Study area

The Republic of Haiti is located on the island of Hispaniola in the Greater Antilles archipelago of the Caribbean Sea, East of Cuba and Jamaica and South of the Bahamas, the Turks, and Caicos islands, and shares the island of Kiskeya with the Dominican Republic. Haiti is the largest country in the Caribbean with a total land area of 27,750 km^2^. Economically, Haiti remains the poorest country in the LAC region and among the poorest countries in the world with a GDP per capita of 1815 USD [35] and ranks 170 out of 189 countries according to the UN’s Human Development Index [36]. Demographically, the current population of Haiti is 11,724,055 and the population density is 414 per km^2^ [37].

### Type of study and data source

This study was cross-sectional, retrospective and used secondary data from the most recent Haiti Demographic and Health Surveys (HDHS) collected between November 2016 and April 2017. The survey was carried out by the Haitian Institute for Children with ICF International providing technical support for the survey through MEASURE DHS. More specifically, the 2016-2017 HDHS collected information on household population and characteristics including information on access to toilets, fertility, marriage, and sexual activity, nutrition, malaria, HIV-AIDS, maternal and child health, adult and childhood mortality, women’s empowerment, domestic violence, and other health-related issues.

### Sampling

HDHS used a stratified two-stage cluster design where in the first stage 450 Enumeration Areas (EA) were selected. In the second stage, a random sample of 13,451 households was drawn from the selected EAs of which 13,405 were successfully interviewed, yielding a response rate of 99.7%. Detailed information regarding the HDHS sampling are published elsewhere [4].

### Definition of variables

#### Dependent variable

In this study, the main outcome of interest was open defecation (OD). The OD variable was coded “yes” if any household practiced open defecation and “no” otherwise.

#### Independent variables

Several variables (place of residence, region, sex of household head, age of household head, household head’s education level, number of household members, household wealth, number of children aged 1-14 years old in the household, number of elderly (aged 65 and above), number of men and women in the household, access to mass media, and marital status) were considered in this study as covariates. These covariates were selected following a literature review on factors found to significantly influence open defecation practice in various studies conducted in developing countries [22, 25, 27, 30, 38–40].

We utilized the existing coding for the place of residence, region, sex of household head, and education level as found in the HDHS *Household Recode* dataset. In the DHS, the place of residence was divided into ‘urban’ and ‘rural’ areas. The region was coded as ‘Aire Métropolitaine de Port-au-Prince’, ‘Reste-Ouest’, ‘Sud-Est’, ‘Nord’, ‘Nord-Est’, ‘Artibonite’, ‘Centre’, ‘Sud’, ‘Grand’Anse’, ‘Nord-Ouest’ and ‘Nippes’. The sex of the household head was coded as ‘male’ and ‘female’. The education level was grouped as ‘no formal education’, ‘primary’, ‘secondary’, and ‘higher’. In the *Household Recode* dataset, the age of the household head, number of household members, and number of children aged 1-14 years old in the household were continuous variables. However, for operational reasons, we decided to recode them. The covariate age of household heads was eventually ranked as follows: ‘less than 25 years’, ‘25-34’, ‘35-44’, ‘45-54’, ‘55-64’, and ‘65 and above’. The number of household members was coded as ‘less than 3’, ‘3-5’, and ‘more than 5’. The covariate number of children aged 1-14 years old in the household was categorized into ‘no children’, ‘one’, ‘2-3’, and ‘4 or more’. Number of elderly (aged 65 and above) was coded as ‘none’, ‘only one’, and ‘two and above’. Number of men and women in the household was divided into ‘fewer women’, ‘equal’, and ‘more women’. Access to mass media was a composite variable that was created by using two variables: access to radio and access to TV. In the HDHS *Household Recode* dataset, each type of mass media was coded as ‘yes’, and ‘no’. After examining the frequency distribution of the responses from the households, we recoded it as ‘yes’ if the household had access to at least one of these mass media, and ‘no’ if the household didn’t have access to any of them.

The household wealth covariate in the DHS is an index of household assets and utilities. To calculate this wealth index, a Principal Component Analysis (PCA) has been used, where questions about household construction materials, water, and sanitation access, and ownership of various assets (eg, radio, TV) are determined at the household level and then individuals are ranked based on the score of the households they live in. Furthermore, the rank positions are used to categorize individuals into five groups: ‘poorest’, ‘poorer’ ‘middle’, ‘richer’, and ‘richest’ [41]. As the wealth index took into account the ‘toilet facilities’ and ‘medias’ covariates, to avoid multicollinearity problems, we created a new wealth index by removing these two covariates while using the PCA approach and keeping the same quintiles. Finally, marital status was described as a three-category variable: ‘never married’, ‘in union’, and ‘divorced/widowed/separated’. We defined a household head ‘in union’ as a household head in a formal marriage or consensual union.

### Data analysis

Statistical analyses were performed with STATA 14 software. Frequency distribution tables were used to draw households’ socioeconomic and demographic profiles. Thereafter, bivariate analysis was conducted using Pearson’s chi-square test to assess whether there existed significant associations between the outcome (OD) and independent variables. Lastly, multivariable analysis was performed using binary logistic regression. In addition, to better explore possible reasons for differences in the prevalence of OD, sub-sample analyzes of the multivariable logistic regression by urban vs. rural, poor vs. non-poor, and low education vs. high education sub-groups were also performed. The model fit was checked with Hosmer–Lemeshow goodness of fit test. Except for the model estimated for the urban area, a good fit was obtained (*P*-value > 0.68). The variance inflation factor (VIF) was used to evaluate potential multicollinearity. The results of the means VIF were below the recommended threshold of 5 [42]. All explanatory variables were included in the multivariate analysis. The results were presented as adjusted odds ratios (AORs), at 95% confidence intervals (CIs). The sample weights (HV005/1,000,000) were applied to get unbiased estimates, according to the DHS guidelines. Furthermore, the survey command (*svy*) in Stata was used to adjust for the complex sampling structure of the data. Statistical significance was set at *P* < 0.05.

### Ethics

This study is based on a secondary analysis of publicly available data (https://dhsprogram.com/data/available-datasets.cfm); therefore, no ethics approval was required from our institutions. Although no permission is required to access these datasets, the corresponding author of this paper sought and obtained on May 3, 2022, the favorable opinion of the Demographic and Health Surveys (DHS) Program for use of the data.

## Results

### Background characteristics of households

The households’ socioeconomic profiles are presented in Table 1. Nearly 6 in 10 households lived in rural areas. Slightly more than 20% of the households came from the Aire Métropolitaine de Port-au-Prince; 16.9% in the Reste Ouest and 15.5% in Artibonite. These three regions account for more than half of the households interviewed. However, Nord-Est (3.3%), Nippes (3.5%), and Grand’Anse (4.2%) are the regions with the lowest proportions of households. Nearly half of the households consisted of three to five members; 27.8% had more than five members, and 23% had less than 3 members. The average household size was 4.3 members (SD ± 2.3). More than 55% of households had no children aged between 1 to 14 years old, 17.3% had one child aged 1 to 14 years old; 21.2% had 2 to 3, and 6% had 4 or more. Also, 54% of households had access to mass media, 35.6% were in the lowest quintiles (poorest/poorer), and 43% were in the highest wealth quintiles (richer/richest). About 55% of household heads were males. Of all household heads that constitute our sample, roughly 25% were aged less than 35 years, of whom 4.3% were young; 21.7% were 35 to 44 years; 20.6% were in the 45-54 age group; 16.8% were 55 to 64 years, and 16.5% were aged 65 or more. In addition, 35% of them had no formal education, 31.6% had a primary education level; 26.8% had a secondary education level, and only 6.4% had higher education. Most of the household heads (66.6%) were in a union, 7.3% had never been married, and 26.1% were divorced or widowed. In addition, in more than three-quarters of the households, there were no elderly (aged 65 years and above). However, 1.9% of the households had one and 4.5% had two or more elderly persons. More than half of the households (51%) had more women than men while in 21% of the households, the number of women was equal to that of men.

**Table 1:** Socio-economic and demographic profiles of households in Haiti

Socio-economic and demographic characteristics	*N*	Percentage
Place of residence		
Urban	5373	40.1
Rural	8032	59.9
Region		
AMPAP	2914	21.7
Reste-Ouest	2261	16.9
Sud-Est	805	6.0
Nord	1295	9.7
Nord-Est	449	3.3
Artibonite	2083	15.5
Centre	958	7.1
Sud	959	7.2
Grand’Anse	560	4.2
Nord-Ouest	657	4.9
Nippes	464	3.5
Sex of household head		
Male	7362	54.9
Female	6043	45.1
Age of household head		
Less than 25 years	570	4.3
25-34	2706	20.2
35-44	2904	21.7
45-54	2762	20.6
55-64	2248	16.8
65 and above	2215	16.5
Education level of household head		
No formal education	4689	35.0
Primary	4238	31.6
Secondary	3589	26.8
Higher	861	6.4
Don’t know	28	0.2
Marital status		
Never married	976	7.3
In union	8920	66.6
Divorced/Widowed	3509	26.1
Household size		
Less than 3	3087	23.0
3-5	6597	49.2
More than 5	3721	27.8
Number of children aged 1-14 years old		
No children	7444	55.5
Only one	2318	17.3
2-3	2838	21.2
4 and above	805	6.0
Number of elderly (aged 65 and above)		
None	10,250	76.5
Only one	2556	19.0
Two and above	599	4.5
Number of men vs. women in the household		
Fewer women	3553	26.5
Equal	3006	22.4
More women	6846	51.1
Access to mass media		
Yes	7245	54.0
No	6160	46.0
Wealth Index		
Poorest	2387	17.8
Poorer	2390	17.8
Middle	2868	21.4
Richer	3349	25.0
Richest	2411	18.0
Total	13,405	100.0

### Prevalence of open defecation practice by socio-economic and demographic characteristics of households

Table 2 includes information on open defecation by selected socio-economic characteristics of households. In Haiti, 25.3% (95% CI: 24.6 - 26.0) of households practiced OD; however this prevalence masks significant geographical, social, and economic disparities. The results indicated that OD was most common in rural areas (35.8%). The practice was most prevalent in ‘Grand’Anse’ region (49.5%), and least prevalent in the ‘Ouest’ (16%). There are also intra-regional disparities. Considering the ‘Ouest’ region, OD practice was most common in the ‘Reste-Ouest’ (26.7%), and least common in the ‘Aire Métropolitaine de Port-au-Prince’ (7.7%) (Table 2).

**Table 2:** Prevalence of open defecation practice by socio-economic and demographic characteristics of households

Socio-economic and demographic characteristics	Open defecation practice		P-value
	Yes N (%)	No N (%)	
Place of residence			
Urban	513 (9.6)	4860 (90.4)	0.000
Rural	2876 (35.8)	5156 (64.2)	
Region			
Aire Métropolitaine de Port-au-Prince	225 (7.7)	2689 (92.3)	0.000
Reste-Ouest	603 (26.7)	1658 (73.7)	
Sud-Est	186 (23.1)	619 (76.9)	
Nord	345 (26.7)	950 (73.3)	
Nord-Est	85 (18.9)	364 (81.1)	
Artibonite	596 (28.6)	1487 (71.4)	
Centre	352 (36.7)	606 (63.3)	
Sud	337 (35.2)	622 (64.8)	
Grand’Anse	277 (49.5)	283 (50.5)	
Nord-Ouest	206 (31.3)	451 (68.7)	
Nippes	178 (38.4)	286 (61.6)	
Sex of household head			
Male	1967 (26.7)	5395 (73.3)	0.000
Female	1422 (23.5)	4621 (76.5)	
Age of household head			
Less than 25 years	151 (26.5)	419 (73.5)	0.000
25-34	581 (21.5)	2125 (78.5)	
35-44	681 (23.5)	2223 (76.5)	
45-54	683 (24.7)	2079 (75.3)	
55-64	633 (28.2)	1615 (71.8)	
65 and above	661 (29.8)	1554 (70.2)	
Education level of household head			
No formal education	1850 (39.5)	2839 (60.5)	0.000
Primary	1161 (27.4)	3077 (72.6)	
Secondary	366 (10.2)	3223 (89.8)	
Higher	99 (1.2)	852 (98.8)	
Don’t know	3 (9.4)	25 (90.6)	
Marital status			
Never married	134 (13.7)	842 (86.3)	0.000
In union	2311 (25.9)	6609 (74.1)	
Divorced/Widowed	945 (26.9)	2564 (73.1)	
Household size			
Less than 3	810 (26.2)	2277 (73.8)	0.283
3-5	1624 (24.6)	4973 (75.4)	
More than 5	955 (25.7)	2766 (74.3)	
Number of children aged 1-14 years old			
No children	1767 (23.7)	5677 (76.3)	0.000
Only one	551 (23.8)	1767 (76.2)	
2-3	787 (27.7)	2051 (72.3)	
4 and above	285 (35.3)	520 (64.7)	
Number of elderly (aged 65 and above)			
None	2496 (24.4)	7755 (75.6)	0.000
Only one	745 (29.2)	1810 (70.8)	
Two and above	148 (24.8)	450 (75.2)	
Number of men and women in the household			
Fewer women	982 (27.6)	2572 (72.4)	0.000
Equal	796 (26.5)	2209 (73.5)	
More women	1611 (23.5)	5234 (76.5)	
Access to mass media			
Yes	910 (12.6)	6335 (87.4)	0.000
No	2480 (40.3)	3680 (59.7)	
Wealth Index			
Poorest	1046 (43.8)	1341 (56.2)	0.000
Poorer	669 (28.0)	1721 (72.0)	
Middle	539 (18.8)	2329 (81.2)	
Richer	677 (20.2)	2672 (79.8)	
Richest	458 (19.0)	1953 (81.0)	
Total	3389 (25.3)	10,016 (74.7)	

The analysis revealed that 23.5 and 26.7% of female and male-headed households practiced OD. Further, OD prevalence was 26.5% among household heads aged less than 25 years, 21.5% for 25-34 years old, 23.5% for 35-44 years old, 24.7% for 45-54 years old, 28.2% for 55-64, and 29.8% for 65 years old or more. As expected, the poorest households, households that had no access to mass media, and households in which the heads had no formal education level practice OD the most (43.8, 40.3, and 39.5%, respectively). Similarly, OD prevalence was much higher among households in which the heads were divorced/widowed (26.9%). We also found that OD was most common in households that had 4 or more children aged between 1 to 14 years old (35.3%) and least common in those there were no children aged between 1 to 14 years old (23.7%). Turning to household size, the difference in prevalence between the categories was very small (less than 3 members: 26.2%; 3-5 members: 24.6%; more than 5: 25.7%). Moreover, OD was most common in households where there was one elderly (29.2%) and almost similar in households with none or two or more elderly (24.4 and 24.8%, respectively). The prevalence of OD was much higher among households with fewer women (27.6%) than those with more women (23.5%). Additionally, chi-square tests showed that except for *household size*, all other covariates had significant associations with OD practice.

### Predictors of OD practice in Haiti

Table 3 shows the results of the first model on predictors of OD practice in Haiti and confirms certain trends observed in Table 2. The findings suggest that *place of residence*, *region*, *sex of household head*, *age of household head*, *education level*, *marital status*, *household size*, *number of children aged 1-14 years old in the household*, *number of elderly*, *number of men and women in the household*, *access to mass media*, and *wealth index* significantly influence OD practice. Compared with those from other regions (all for whom AOR < 1), households from Grand’Anse were more likely to practice OD. The results indicate that households living in rural areas were two times more likely to practice OD (AOR = 2.01; 95% CI: 1.76 – 2.30) than those in urban areas. We also found that households consisting of less than three members were 1.4 times more likely (AOR = 1.40; 95% CI: 1.21 - 1.62) to practice OD than households with more than five members. Similarly, households that had one child aged 1-14 years old (AOR = 0.81; 95% CI: 0.66 – 0.99) or none (AOR = 0.74; 95% CI: 0.61 – 0.88) were less likely to practice OD than those who had 4 children aged 1-14 years old or more.

**Table 3:** Logistic regression estimates for OD practice, with adjustment for selected covariates

Socio-economic and demographic characteristics	P-Value	Adjusted Odds Ratio (AOR)	95% CI
Place of residence			
Rural	0.000	2.01	1.76 – 2.30
Urban = Ref.			
Region			
Aire Métropolitaine de Port-au-Prince	0.000	0.39	0.31 – 0.51
Reste-Ouest	0.000	0.46	0.38 – 0.55
Sud-Est	0.000	0.35	0.28 – 0.42
Nord	0.000	0.53	0.43 – 0.64
Nord-Est	0.000	0.31	0.25 – 0.38
Artibonite	0.000	0.43	0.36 – 0.52
Centre	0.000	0.52	0.43 – 0.63
Sud	0.000	0.63	0.52 – 0.77
Nord-Ouest	0.000	0.49	0.40 – 0.59
Nippes	0.000	0.68	0.56 – 0.83
Grand’Anse = Ref.			
Sex of household head			
Female	0.000	0.74	0.67 – 0.81
Male = Ref.			
Age of household head			
Less than 25 years	0.000	3.06	2.23 – 4.20
25-34	0.000	2.30	1.82 – 2.90
35-44	0.000	1.77	1.42 – 2.20
45-54	0.015	1.30	1.05 – 1.59
55-64	0.165	1.15	0.94 – 1.41
65 and above = Ref.			
Education level of household head			
No formal education	0.000	23.04	13.34 – 39.80
Primary	0.000	13.00	7.54 – 22.34
Secondary	0.000	5.59	3.23 – 9.64
Higher = Ref.			
Marital status			
Never married	0.002	0.68	0.53 - 0.87
In union	0.026	0.88	0.78 - 0.98
Divorced/Widowed = Ref.			
Household size			
Less than 3	0.000	1.40	1.21 – 1.62
3-5	0.003	1.18	1.06 – 1.32
More than 5 = Ref.			
Number of children aged 1-14 years old			
No children	0.001	0.74	0.61 – 0.88
Only one	0.040	0.81	0.66 – 0.99
2-3	0.177	0.88	0.73 – 1.06
4 or more = Ref.			
Number of elderly (aged 65 and above)			
Only one	0.330	1.09	0.92 – 1.28
Two and above	0.198	0.84	0.65 – 1.09
None = Ref.			
Number of men vs. women in the household			
Equal	0.178	0.92	0.81 – 1.04
More women	0.068	0.91	0.82 – 1.01
Fewer women = Ref.			
Access to mass media			
No	0.000	2.35	2.14 – 2.58
Yes = Ref.			
Wealth Index			
Poorest	0.000	2.13	1.83 – 2.47
Poorer	0.000	1.64	1.41 – 1.91
Middle	0.059	1.16	0.99 – 1.36
Richer	0.940	1.01	0.87 – 1.17
Richest = Ref.			

Pearson chi2 = 11,517.59

Prob > Chi2 = 0.9973

Mean VIF = 3.32

Pseudo R2 = 0.1801

Poor households had 1.6 to 2.1 greater odds (AOR = 2.13; 95% CI: 1.83 – 2.47; AOR = 1.64; 95% CI: 1.41 – 1.91) of resorting to OD than the richest households. Households that didn’t have access to mass media were 2.4 times more likely to practice OD (AOR = 2.35; 95% CI: 2.14 – 2.58) than households that have access to mass media. Households in which the head had no formal education were 23 times more likely (AOR = 23.04; 95% CI: 13.34 – 39.80) to practice OD than those in which the head had a higher education level. Similarly, households in which the head had primary or secondary education levels were at least 5.6 times more likely to defecate in the open than those in which the head had a higher education level. The likelihood of OD was significantly higher among younger household heads (AOR = 3.06; 95% CI: 2.23– 4.20) compared to those aged 65 years or more. The same trend was observed for the age groups 25-34, 35-44, 45-54, and 55-64. Finally, the results revealed that households in which the head had never married (AOR = 0.68; 95% CI: 0.53 - 0.87) and in union (AOR = 0.88; 95% CI: 0.78 - 0.98) were less likely to practice OD as compared with those in which the head was divorced/widowed.

Tables 4, 5 and 6 present comparisons between rural/urban areas, poor/non-poor households, and low-educated/high-educated household heads. Indeed, we observed that there were no great differences between the results of these tables and those of Table 3.

**Table 4:** Logistic regression model: urban vs. rural comparisons, with adjustment for selected covariates

Sociodemographic Characteristics	Urban		Rural	
	P-Value	AOR (95% CI)	P-Value	AOR (95% CI)
Region				
Aire Métropolitaine de Port-au-Prince	0.000	0.23 (0.16 - 0.33)		
Reste-Ouest	0.000	0.35 (0.20 - 0.61)	0.000	0.51 (0.41 - 0.63)
Sud-Est	0.005	0.42 (0.23 - 0.77)	0.000	0.38 (0.30 - 0.47)
Nord	0.000	0.25 (0.16 - 0.39)	0.000	0.64 (0.51 - 0.80)
Nord-Est	0.000	0.08 (0.05 - 0.13)	0.000	0.41 (0.32 - 0.53)
Artibonite	0.000	0.13 (0.08 - 0.21)	0.000	0.55 (0.45 - 0.67)
Centre	0.000	0.14 (0.08 - 0.27)	0.000	0.64 (0.52 - 0.78)
Sud	0.006	0.49 (0.30 - 0.82)	0.000	0.68 (0.56 - 0.84)
Nord-Ouest	0.000	0.19 (0.12 - 0.33)	0.000	0.59 (0.48 - 0.73)
Nippes	0.595	1.15 (0.69 - 1.92)	0.000	0.67 (0.54 - 0.83)
Grand’Anse = Ref.				
Sex of household head				
Female	0.000	0.63 (0.51 - 0.80)	0.000	0.78 (0.71 - 0.87)
Male = Ref.				
Age of household head				
Less than 25 years	0.000	12.18 (6.01 - 24.73)	0.000	2.09 (1.46 - 2.99)
25-34	0.000	5.21 (2.91 - 9.32)	0.000	1.92 (1.49 - 2.49)
35-44	0.000	4.32 (2.47 - 7.55)	0.002	1.45 (1.14 - 1.84)
45-54	0.001	2.43 (1.42 - 4.14)	0.231	1.15 (0.92 - 1.44)
55-64	0.036	1.82 (1.04 - 3.17)	0.572	1.07 (0.86 - 1.33)
65 and above = Ref.				
Education level of household head				
No formal education	0.000	37.15 (15.64 - 88.25)	0.000	16.75 (8.12- 34.57)
Primary	0.000	17.52 (7.52 - 40.82)	0.000	9.90 (4.81 - 20.39)
Secondary	0.000	5.90 (2.53 - 13.75)	0.000	4.81 (2.33 - 9.97)
Higher = Ref.				
Marital status				
Never married	0.016	0.51 (0.29 - 0.88)	0.109	0.79 (0.59 - 1.05)
In union	0.912	1.02 (0.77 - 1.33)	0.021	0.86 (0.76 - 0.98)
Divorced/Widowed/Separated = Ref.				
Household size				
Less than 3	0.017	1.58 (1.08 - 2.29)	0.000	1.34 (1.14 - 1.58)
3-5	0.160	1.23 (0.92 - 1.64)	0.011	1.17 (1.04 - 1.32)
More than 5 = Ref.				
Number of children 1-14 years old				
No children	0.035	0.58 (0.35 - 0.96)	0.005	0.76 (0.62 - 0.92)
Only one	0.128	0.65 (0.38 - 1.13)	0.074	0.82 (0.66 - 1.02)
2-3	0.215	0.72 (0.43 - 1.21)	0.237	0.89 (0.73 - 1.08)
4 and above = Ref.				
Number of elderly (aged 65 and above)				
One	0.040	1.53 (1.02 - 2.29)	0.879	1.01 (0.85 - 1.21)
Two and above	0.359	1.45 (0.66 - 3.21)	0.088	0.79 (0.59 - 1.04)
None = Ref.				
Number men vs. women in the household				
Equal	0.646	0.93 (0.68 - 1.27)	0.152	0.90 (0.79 - 1.04)
More women	0.012	0.72 (0.55 - 0.93)	0.314	0.94 (0.84 - 1.06)
Fewer women = Ref.				
Access to mass media				
No	0.000	3.36 (2.71 - 4.18)	0.000	2.12 (1.95 - 2.35)
Yes = Ref.				
Wealth Index				
Poorest	0.000	3.12 (2.17 - 4.51)	0.000	2.18 (1.83 - 2.58)
Poorer	0.442	0.86 (0.58 - 1.27)	0.000	1.87 (1.56 - 2.23)
Middle	0.451	1.12 (0.83 - 1.52)	0.059	1.19 (0.99 - 1.44)
Richer	0.266	0.85 (0.63 - 1.13)	0.568	1.05 (0.88 -1.25)
Richest = Ref.				
	Pearson chi2 = 4203.95		Pearson chi2 = 7804.82	
	Prob > Chi2 = 0.0375		Prob > Chi2 = 0.6885	
	Mean VIF > 5		Mean VIF = 3.69	
	Pseudo R2 = 0.2384		Pseudo R2 = 0.1013

**Table 5:** Logistic regression model: low education vs. high education comparisons, with adjustment for selected covariates

Sociodemographic Characteristics	Low education		High education	
	*P*-Value	AOR (95% CI)	*P*-Value	AOR (95% CI)
Place of residence				
Rural	0.000	2.04 (1.76 - 2.38)	0.000	2.59 (1.96 - 3.43)
Urban = Ref.				
Region				
Aire Métropolitaine de Port-au-Prince	0.000	0.51 (0.38 - 0.68)	0.000	0.16 (0.09 - 0.27)
Reste-Ouest	0.000	0.58 (0.47 - 0.72)	0.000	0.18 (0.11 - 0.29)
Sud-Est	0.000	0.39 (0.32 - 0.49)	0.000	0.20 (0.12 - 0.35)
Nord	0.000	0.63 (0.51 - 0.78)	0.000	0.18 (0.09 - 0.32)
Nord-Est	0.000	0.36 (0.29 - 0.46)	0.000	0.16 (0.08 - 0.29)
Artibonite	0.000	0.51 (0.42 - 0.62)	0.000	0.26 (0.16 - 0.42)
Centre	0.000	0.60 (0.49 - 0.73)	0.000	0.30 (0.18 - 0.51)
Sud	0.002	0.72 (0.59 - 0.89)	0.000	0.31 (0.19 - 0.52)
Nord-Ouest	0.000	0.56 (0.46 - 0.69)	0.000	0.27 (0.17 - 0.43)
Nippes	0.004	0.73 (0.59 - 0.91)	0.004	0.48 (0.29 - 0.78)
Grand’Anse = Ref.				
Sex of household head				
Female	0.000	0.82 (0.74 - 0.91)	0.010	0.72 (0.56 - 0.92)
Male = Ref.				
Age of household head				
Less than 25 years	0.000	2.06 (1.42 - 2.99)	0.001	6.58 (2.27 - 19.06)
25-34	0.001	1.55 (1.21 - 1.99)	0.004	4.35 (1.60 - 11.82)
35-44	0.011	1.35 (1.07 - 1.70)	0.037	2.91 (1.07 - 7.94)
45-54	0.277	1.13 (0.91 - 1.40)	0.342	1.64 (0.59 - 4.58)
55-64	0.393	1.09 (0.89 - 1.36)	0.626	1.32 (0.43 - 4.09)
65 and above = Ref.				
Marital status				
Never married	0.201	0.81 (0.58 - 1.13)	0.002	0.46 (0.28 - 0.74)
In union	0.018	0.86 (0.77 - 0.97)	0.172	0.77 (0.53 - 1.12)
Divorced/Widowed/Separate = Ref.				
Household size				
Less than 3	0.000	1.47 (1.26 - 1.72)	0.773	1.06 (0.71 - 1.61)
3-5	0.004	1.19 (1.06 - 1.34)	0.432	1.14 (0.83 - 1.57)
More than 5 = Ref.				
Number of children 1-14 years old				
No children	0.001	0.72 (0.60 - 0.88)	0.109	0.63 (0.36 - 1.11)
Only one	0.014	0.76 (0.61 - 0.95)	0.403	0.77 (0.42 - 1.41)
2-3	0.241	0.89 (0.73 - 1.08)	0.166	0.67 (0.38 - 1.18)
4 and above = Ref.				
Number of elderly (aged 65 and above)				
One	0.617	1.05 (0.88 - 1.25)	0.591	1.12 (0.74 - 1.71)
Two and above	0.186	0.83 (0.64 - 1.09)	0.549	0.69 (0.22 - 2.25)
None = Ref.				
Number men vs. women in the household				
Equal	0.143	0.90 (0.79 - 1.03)	0.957	0.99 (0.70 - 1.39)
More women	0.073	0.90 (0.81 - 1.01)	0.319	0.87 (0.66 - 1.15)
Fewer women = Ref.				
Access to mass media				
No	0.000	2.35 (2.13 - 2.60)	0.000	2.85 (2.27 - 3.58)
Yes = Ref.				
Wealth Index				
Poorest	0.000	2.19 (1.87 - 2.56)	0.001	1.94 (1.31 - 2.89)
Poorer	0.000	1.93 (1.63 - 2.27)	0.041	0.64 (0.41 - 0.98)
Middle	0.022	1.22 (1.03 - 1.44)	0.193	0.77 (0.52 - 1.14)
Richer	0.831	0.98 (0.84 - 1.15)	0.793	0.95 (0.65 - 1.38)
Richest = Ref.				
	Pearson chi2 = 7849.37		Pearson chi2 = 3314.71	
	Prob > Chi2 = 0.8006		Prob > Chi2 = 0.7161	
	Mean VIF = 2.83		Mean VIF = 3.75	
	Pseudo R2 = 0.1054		Pseudo R2 = 0.1909

**Table 6:** Logistic regression model: poor vs. non-poor comparisons, with adjustment for selected covariates

Sociodemographic Characteristics	Poor		Non-poor	
	P-Value	AOR (95% CI)	P-Value	AOR (95% CI)
Place of residence				
Rural	0.000	2.44 (1.92 - 3.11)	0.000	1.91 (1.63 - 2.24)
Urban = Ref.				
Region				
Aire Métropolitaine de Port-au-Prince	0.000	0.41 (0.25 - 0.65)	0.000	0.33 (0.24 - 0.45)
Reste-Ouest	0.000	0.48 (0.36 - 0.63)	0.000	0.37 (0.28 - 0.50)
Sud-Est	0.000	0.30 (0.22 - 0.41)	0.000	0.31 (0.23 - 0.42)
Nord	0.000	0.58 (0.44 - 0.76)	0.000	0.43 (0.32 - 0.58)
Nord-Est	0.000	0.39 (0.29 - 0.51)	0.000	0.20 (0.14 - 0.28)
Artibonite	0.000	0.47 (0.37 - 0.60)	0.000	0.36 (0.27 - 0.47)
Centre	0.000	0.55 (0.43 - 0.71)	0.000	0.44 (0.33 - 0.60)
Sud	0.783	0.96 (0.74 - 1.26)	0.000	0.40 (0.30 - 0.54)
Nord-Ouest	0.000	0.53 (0.41 - 0.68)	0.000	0.42 (0.32 - 0.56)
Nippes	0.066	0.77 (0.59 - 1.02)	0.000	0.55 (0.41 - 0.74)
Grand’Anse = Ref.				
Sex of household head				
Female	0.000	0.69 (0.60 - 0.80)	0.000	0.77 (0.68 - 0.88)
Male = Ref.				
Age of household head				
Less than 25 years	0.000	3.03 (1.89 - 4.86)	0.000	3.20 (2.09 - 4.90)
25-34	0.000	2.24 (1.59 - 3.16)	0.000	2.46 (1.79 - 3.39)
35-44	0.002	1.67 (1.21 - 2.30)	0.000	1.92 (1.42 - 2.59)
45-54	0.028	1.41 (1.04 - 1.91)	0.110	1.26 (0.95 - 1.68)
55-64	0.421	1.13 (0.84 - 1.52)	0.218	1.19 (0.90 - 1.57)
65 and above = Ref.				
Education level of household head				
No formal education	0.000	38.32 (15.48 - 94.88)	0.000	14.73 (7.40 - 29.33)
Primary	0.000	23.45 (9.51 - 57.85)	0.000	7.87 (3.98 - 15.56)
Secondary	0.000	9.41 (3.80 - 23.33)	0.000	3.45 (1.74 - 6.84)
Higher = Ref.				
Marital status				
Never married	0.000	0.49 (0.34 - 0.72)	0.394	0.87 (0.63 - 1.20)
In union	0.000	0.72 (0.61 - 0.86)	0.893	1.01 (0.86 - 1.18)
Divorced/Widowed/Separated = Ref.				
Household size				
Less than 3	0.000	1.50 (1.21 - 1.87)	0.022	1.27 (1.04 - 1.55)
3-5	0.005	1.26 (1.07 - 1.48)	0.281	1.09 (0.93 - 1.27)
More than 5 = Ref.				
Number of children 1-14 years old				
No children	0.028	0.75 (0.58 - 0.97)	0.025	0.74 (0.58 - 0.96)
Only one	0.164	0.81 (0.61 - 1.09)	0.185	0.83 (0.62 - 1.10)
2-3	0.858	0.98 (0.75 - 1.27)	0.138	0.82 (0.63 - 1.07)
4 and above = Ref.				
Number of elderly (65 and above)				
One	0.743	1.04 (0.82 - 1.33)	0.299	1.13 (0.90 - 1.40)
Two and above	0.334	0.83 (0.57 - 1.21)	0.354	0.85 (0.59 - 1.21)
None = Ref.				
Number men vs. women in households				
Equal	0.244	0.90 (0.75 - 1.08)	0.421	0.93 (0.78 - 1.11)
More women	0.214	0.91 (0.78 - 1.06)	0.152	0.90 (0.78 - 1.04)
Fewer women = Ref.				
Access to mass media				
No	0.000	2.10 (1.82 - 2.41)	0.000	2.52 (2.22 - 2.85)
Yes = Ref.				
	Pearson chi2 = 4061.72		Pearson chi2 = 6217.40	
	Prob > Chi2 = 0.9964		Prob > Chi2 = 0.9649	
	Mean VIF = 3.30		Mean VIF = 3.67	
	Pseudo R2 = 0.1738		Pseudo R2 = 0.1355	

## Discussion

This paper is the first to focus on the prevalence and factors influencing OD in Haiti. Data used in this study are retrieved from the 2016-2017 HDHS, a national representative survey conducted by the Haitian Institute for Children in collaboration with ICF International. Further, to achieve the main of the study, we used scientifically validated methods (descriptive and multivariate analysis).

We found that the overall proportion of households practicing OD in Haiti was estimated at 25.3% (95% CI: 24.6 - 26.0), seven times higher than in the Dominican Republic (3.4%) [43] and comparable to the average prevalence of OD among households in sub-Saharan countries (22.5%) [27]. This relatively high prevalence of OD practice in Haiti is concerning, particularly due to the significant high risk of disease transmission it poses in the country. Therefore, gaining an understanding of the factors related to the behavior is imperative to eliminate the practice. Results from the present study suggest that OD was significantly associated with several socio-economic and demographic factors *the age of the household head*, *sex of the household head*, *household size*, *number of children aged 1-14 years old in the household*, *education level*, *wealth index*, *access to mass media*, *place of residence*, and *region*. To reduce the high prevalence of OD in Haiti, policymakers should consider these factors. Our findings revealed that poor households were more likely to practice OD. This finding is consistent with studies in Africa [22, 25, 27, 29, 38] and Indonesia [39]. A plausible explanation for the observed association is that poor households are more likely to face financial constraints to build a toilet [30, 44, 45] or construct simple toilets which fill up quickly and are prone to collapse when subjected to heavy rains or floods [38]. Also, those with rudimentary latrines may be financially constrained to upgrade them [46] resulting in slippages [26, 39].

As was expected, households with an educated head were associated with a higher likelihood of not defecating in the open than those with uneducated heads, that is, the odds of defecating in the open decreased as the educational level increased. This evidence corroborates the association found in past studies [22, 27, 40, 47]. Indeed, education enables an understanding of improved sanitation, the effects of defecating in the open as well as the relevance of owning a toilet [22]. In addition, a higher education level could affect household income, thereby providing the means to own a toilet [40].

Results found that having no access to mass media strongly predicts OD. This is an important finding since to our knowledge no previous research has tested this variable in OD prediction. Numerous studies have shown that mass media remains a vital source of information and can raise awareness, increase knowledge levels, and influence household behaviors and attitudes [48, 49]. Households that are exposed to mass media are more likely to be informed about the effects of OD which provides a better way to understand the benefits of using a toilet [40]. In Haiti, as in many countries, the government usually uses mass media campaigns for sanitation promotion [50]. However, to reach households living in rural areas with limited access to electricity, the government would better mobilize community-based communication strategies, including local existing social networks and interpersonal communication [40].

Similar to other studies [22, 24, 40], our findings suggest that place of residence was a significant predictor of OD and that households in a rural environment are significantly more likely than those in urban settings to defecate in the open. This probably reflects different factors. First, Haitian rural households primarily engaged in agriculture [51]. The majority of their time is spent working in the agricultural fields. In such a context, even if they have access to a public latrine, they might not use it which may become a norm in rural areas [21]. Second, factors such as unequal distribution of resources and limited access to information and sanitary infrastructure that characterize rural settings lead to the practice of OD [27]. In Haiti, the level of poverty is much higher in rural areas (74.9%) than in urban areas (40.6%) [52]. Third, in urban areas, it is seen as socially shameful to defecate in the open [53]. Lastly, households in rural areas have less access to mass media than their counterparts [4], hence less exposed to information that can influence OD practice as well as local beliefs and behaviors [21].

The geographical region was significantly associated with OD. The study noticed that households in Grand-Anse were more likely to defecate in the open than households from other regions. Certainly, Grand-Anse is one of the poorest regions in Haiti [52]. The poverty rate in this department was 79.6%. Given the financial handicaps, many households in this region could not consider building a toilet facility a priority. Additionally, Grand-Anse was severely struck by hurricane Matthew in 2016 [54, 55], i.e. 1 month preceding the 2016-2017 HDHS. More than 30,000 houses were destroyed or heavily damaged in the region as well as sanitation infrastructure, which could also explain the greater odds of OD practice in this department compared to others [56].

OD practice is significantly influenced by gender in Haiti. Indeed, households headed by women were less likely to defecate in the open than those headed by men. In line with a study in India [24], this result could be partly attributed to the residential factor. For instance, 44% of households headed by women are urban compared to 37% for those headed by men. As mentioned above, OD is most common in rural areas.

Results further noted that OD practice increased with a decrease in the age of the household head. This finding is supported by previous studies [25, 27]. Since unemployment has increased considerably in younger age groups in Haiti during the last decades [51, 57, 58], youth-headed households were more likely to be poor, affecting their capacity to meet the cost of catering for basic needs including building toilets [22, 59, 60].

The marital status of the household head was significantly associated with OD practice with higher odds of OD where the household head was divorced or widowed. A study conducted in India is in line with our findings [24]. This finding may be accounted for by the fact that our sample of divorced/widowed household heads contains approximately three-quarters of females. Several studies have suggested that divorced/widowed Haitian women are more prone to economic shocks [61–63], and therefore households that they lead might not have access to toilets.

Household size was a significant predictor of OD. Households with less than three members had greater odds to practice OD than households that consisted of three to five members and more than five members. Our result is in concordance with findings in rural China [64] and East Africa [65]. This finding may be partly accounted for by the fact that the majority (65%) of households with heads aged 65 or more are larger-sized households (three to five or six plus members). Evidence shows that the presence of the elderly in households may reduce OD [66] as older people very often have mobility issues, and thus may have great difficulty moving to defecate in the open, especially in the dark [67]. To prevent risks (harassment, assaults, and attacks by animals), households with elderly members would have a further reason to take the necessary steps to build their toilets [68]. On the other hand, 30% of households with less than three members were headed by individuals aged under 35, compared to 12% of households consisting of more than five members. As already discussed, younger generations are particularly affected by the economic crisis in terms of employment [51, 57, 58], which would impact significantly the level of hygienic comfort of the households they lead.

Households with 4 children aged 1-14 years old or more are more likely to defecate in the open than those with no children aged 1-14 years old or one child aged 1-14 years. The large majority of Haitian households with 4 children aged 1-14 years old or more live in rural areas, while more than 40% of households where there are no children aged 1-14 years old or only child aged 1-14 years old live in urban areas. Given that OD practice is mainly a rural issue, this may explain this association.

### Study strengths and limitations

The main strength of this study is the use of a nationally representative survey, therefore our findings can be generalized to all households in Haiti. Moreover, the study results shed light on the factors influencing OD in Haiti which provides invaluable information for interventions. It adds to the literature by including access to mass media as an important predictor of OD practice. Nevertheless, this study is not free of limitations. Because having to defecate openly infringes on human safety and dignity, it may be difficult for some households to report the practice resulting in underreporting which could lead to an underestimation of the phenomenon in Haiti. Furthermore, the cross-sectional nature of the data limits our understanding of causal inferences. However, these limitations do not invalidate our results.

## Conclusion

Although Haiti has approved Sustainable Development Goals including target 6.2, the reduction of OD prevalence and the achievement of improved sanitation remain unsatisfactory. The results showed that one in every four Haitian households engaged in open defecation despite concerted efforts to eradicate the practice from the Haitian government and their partners. The factors influencing OD were age and sex of household head, household size, number of children aged 1-14 years old in the household, education level, wealth index, access to mass media, place of residence, and region. Consequently, to accelerate the elimination of open defecation by 2030 and therefore achieve sustainable open defecation-free status, the government of Haiti and its partners should reinforce their efforts while taking into consideration these factors. Particularly, they would better target rural households while using community-based and interpersonal communication strategies. Policy-makers should pay special attention to the socioeconomic situation of the households. The poor households in urban areas live generally in houses with not enough space for individual toilets. Community-based toilets could help reduce open defecation. Conversely, poor households in rural areas live in dispersed habitats with no sanitation system near them. Possible interventions, in this case, are to subsidize individual toilets, in addition to good sanitation awareness. Interventions should also prioritize households headed by women and young people, two underpriviledged socioeconomic groups in Haiti.

## Data Availability

The dataset used in this study is available on the following repository: https://dhsprogram.com/data/dataset/Haiti_Standard-DHS_2016.cfm?flag=0.
